# Co‐Designing Qualitative Interview Methods With Young Children and Parents: Drawing, Pictures, and Puppets

**DOI:** 10.1111/hex.70942

**Published:** 2026-10-23

**Authors:** Arwa Katab, Bronia Arnott, Timothy Cheetham, Olivia Craw, Niina Kolehmainen

**Affiliations:** ^1^ Population Health Science Institute Newcastle University Newcastle UK; ^2^ Institute of Translational and Clinical Research Newcastle University Newcastle UK; ^3^ Child Health Outcomes Research at Leeds (CHORAL) University of Leeds Leeds UK

**Keywords:** co‐design, participatory methods, Qualitative research, school transition

## Abstract

**Introduction:**

Children have the right to freedom of expression regarding topics important to them via any communication medium.^1^ Conducting research *on* as opposed to *with* young children fails to fulfil their rights, promoting misconceptions that children are incapable of providing meaningful data. The start of formal education presents many challenges for young children; very few studies have involved children as active participants sharing their experiences. This co‐design study aimed to produce, through the involvement of young children, methods for a creative interview package to be used with 4–5‐year‐olds to discuss the challenges of starting formal education.

**Methods:**

Six individual co‐design sessions, and three user‐testing interviews with young children (aged 3‐5 years), alongside one group co‐design session with older children (aged 6–12 years), were used to develop and refine methods. Pictures and photo‐prompts, drawing activities, and the use of a toy puppet were explored within the sessions, collecting children's verbal feedback and recommendations, as well as the researcher's reflective notes and recommendations from parents. The resulting methods were integrated into a four‐stage framework (Exploration, Adaptation, Confirmation, and Evaluation) to curate the final interview methods.

**Results:**

Barriers to children's engagement were identified with some participatory activities, for example cultural and family belief systems that discouraged self‐portrait drawings within some communities. Children also engaged more in the interview once they had built a rapport with the puppet, and not only a rapport with the researcher. Pre‐made images were sufficient in obtaining more detailed discussion of the children's experiences.

**Conclusion:**

This study resulted in the first interview methods package for young children which has been co‐designed with young children. The study demonstrates that young children can be active contributors in designing research if provided with the opportunity to do so.

**Patient or Public Contribution:**

Children, parents and teachers provided input and shaped the design of this study.

## 1 Introduction

The United Nations’ Convention on the Rights of the Child protects and promotes children's voices on topics relevant to them, necessitating that their views must be heard, respected and taken seriously [1]. It is now widely accepted that children have the right to be included in all aspects of research, from co‐designers, that is, collaboratively develop, with shared power in designing and conducting research [2, 3], to actively participating in generating, interpreting and implementing its findings [4]. Research studies and methods can be tailored towards children's needs via insights proposed by children themselves [5, 6], and findings from research with children can inform policies and practices which impact their daily lives.

Participatory research with the youngest children however, wherein they help design a study, is relatively scarce within health research [3, 7, 8]. Though there is a growing recognition of the importance of children's voices in research, it has often been accompanied with the involvement of older children [5]. There is an increasing body of literature on ways to include older children and young people in designing and undertaking research [5, 8]. However, involvement of younger children, and frameworks for doing this well, remains limited [8]. In our experience, compared to research with older children, methodological, practical and ethical considerations for doing research *with* (rather than *on*) children under 5‐years‐old continues to be viewed as too challenging, and the belief that young children cannot make a credible contribution remains persistent [8]. Additional time and ethical considerations related to working with young children can further prevent their involvement and participation [8, 9]. Yet a large percentage of funding acquired for health‐related research is wasted due to poor quality research designs and discrepancies between researcher's goals and the public's needs [10]. A requirement for children's involvement during the research development phase is warranted to subside this issue. This present paper seeks to challenge these views by demonstrating 3–5‐year‐olds ability to develop qualitative interview methods within a co‐design study for subsequent use in research with young children. Throughout this paper, *young children* will relate to those 3‐5 years old.

Previous research has shown that child‐friendly participatory methods, such as drawings and puppets [11], can facilitate children's expression of their experiences [12, 13], yet these methods do not guarantee its suitability for all children [14]. Einarsdottir et al [14] observed that some children criticised their drawing abilities, whilst Harrison and Murray [15] observed 4–5‐year‐olds feeling bored during their picture‐elicited interviews. There is a clear gap, and a need for more work, in research methods designed *by* young children *for* young children.

Lundy [16] proposed four elements (space, voice, audience, and influence) to consider when involving young children at various stages of research. *Space* considers the environment as it could affect the integrity of data collected. Early years settings, for example, may prevent children declining participation as they may believe the research activity is schoolwork [14]. Building rapport and empowering children as the experts of their experiences can create safe spaces, supporting children's ability to express their opinions [17, 18]. Lundy [16] further suggested providing methods (tools) to facilitate children's expression (*voice*). Understanding how young children connect with the world, such as through play [19], and how they utilise these tools to express themselves, helps children share their opinions. The appropriate *audience* (e.g., researchers) should then record and make changes based on children's recommendations [16]. Acting on the children's suggestions implements the final element, *influence* [16]. Meeting these four elements ensures young children's involvement honours children's right of expression and views to be taken seriously [1].

This present study aimed to develop a qualitative interview package for use in research with young children, for example research focussing on the transition into formal education. The emphasis was on working with young children themselves to co‐design the package, with the intention of addressing barriers within research methods that could limit young children's voices and engagement.

Creative participatory methods, namely the use of puppets, drawings and pictures, were explored within this study to address the methodological, practical and ethical considerations when interviewing young children. The activities that we co‐designed were developed with the aim to improve the quality of interviews with young children, specifically to build a rapport with them [20], manage the power imbalance between the researcher and child (creating a safe *space*) [16, 18, 21], ensure young children understand the purpose of the study and what will be involved, enable them to express themselves (*voice*) [16, 18], and collect ongoing assent throughout their participation (*space*) [4, 16, 18].

## 2 Methods

This study adopted a co‐design approach wherein children, parents and teachers were co‐design contributors who identified barriers within the research methods that could limit young children's voices and their enjoyment of the activities [22, 23]. This study formed a part of a doctoral research programme of the first author, the overarching purpose of which was to identify ways to support young children's health and wellbeing during the transition to formal education using child‐appropriate research methods.

Working with the researcher, co‐design contributors identified barriers within the research methods that could limit young children's voices and their enjoyment of the activities. Solutions to identified problems were also discussed to meet the needs of the target population (young children) [3, 22, 23], such as how to ensure the methods were engaging and fun for the children. The co‐design process involved trialling different prototypes of the methods and seeking contributor feedback to refine the design of the methods, specifically improve the experience of the participatory methods for the young children [3, 22, 23], whilst meeting the aims of the study to identify and address barriers to young children's participation.

Individual co‐design sessions with young children were prioritised in the first instance to ensure each child's recommendations could be observed and recorded. A key criterion of conducting individual sessions was to be guided by the child's preferences, for example, how they preferred to interact with the chosen method, to implement changes to subsequent sessions. While the primary aim of the study was to understand children's experiences, parents’ perspectives were sought to provide supplementary insights and to support ethical, family‐centred research practices. Some comments were sought from teachers, although this was limited to mainly online interactions (e.g., emails) due to teachers’ busy scheduling.

A group session with older children (6–12‐year‐old) was reserved for the later stages of co‐designing the interview protocol, and this focussed on confirming the appropriateness of the methods. The last stage of the co‐design involved individual user‐tester interviews with child participants to test the final version of the methods. Together, these factors strengthen the impact of the children's contributions to evolving iterations and creative design from one session to the next. Minimal resources (time and money), with young children's development and ethical considerations in mind, was an additional key design principle.

## 3 Materials

Drawings, pictures and puppets were used in this study as they are developmentally appropriate techniques commonly used with young children, enabling the provision of an inclusive *space* for engagement and supporting the expression of children's *voice* [16]. These methods could then be integrated into an interview methods package. Materials were selected so as not to be resource intensive (e.g., expensive or difficult to transport) to maximise accessibility and future transferability across contexts. The drawing activities consisted of A4 white paper, colouring pencils, a graphite pencil, and a rubber, with no restrictions placed on their use to avoid limiting the child's creativity, engagement, or enjoyment [24]. These materials were provided during the in‐person sessions only, online sessions involved alternative activities. Later versions of the drawing activity consisted of an A3 sheet showing an incomplete drawing of two school buildings. Drawing activities can foster rapport with young children through the familiarity of the activity [12, 14]. Likewise, drawings can ease children into sensitive topics and allow them to communicate more easily than solely through words [12].

Pictures involved premade images from Murray and Harrison's [25] Pictural School Stress and Wellbeing measure which consists of nine‐line drawings of children experiencing potential challenges in school (e.g., going to the bathroom alone). Picture‐related sessions also involved photos of the participating children on their first day of school provided by parents. Children producing their own photographs can reduce power imbalance and encourage collaboration between the child and researcher [26]. However, when discussing sensitive subjects, or in settings that are constrained by ethical, practical or safeguarding considerations, such as school environments wherein children may not be permitted to capture photographs of their environments, researcher‐produced images can be used instead to preserve an inclusive participatory *space* [27].

For the puppet sessions, three versions of puppets were used; the first puppet was a female doll, 30 cm in size, with black curly hair and tanned skin. Two identical multi‐coloured snake puppets were also used in this session. The final selected puppet, Mochi, was a soft fluffy orange monster with its orange fluff covering its face and body and was small in size (30 cm in length). Mochi's left arm could be controlled with a detachable arm‐control rod, as well as its mouth and head to exhibit visual cues of listening (e.g., head‐nods) [28], and thinking (e.g., scratching their head). Using puppet‐elicited interviews can help manage the power imbalance between children and researchers by positioning the puppet as the unknowledgeable interviewer, supporting children's *voices* by reducing the pressure on the children to provide ‘correct’ answers and encourages more authentic responses [17, 29].

### 3.1 Setting and Participants

This study was part of a wider PhD project exploring young children's experiences of the transition into formal education (i.e., reception) in the North‐East of England between 2021 and 2022. The target population for this co‐design study were parents and young children (3‐5 years old) who were experiencing the first year of formal education, had recently experienced this (i.e., their child was now in Year 1), or were anticipating the transition. Teachers working with children in reception, or had recently worked with them (e.g., trainee teachers) were also involved in developing the methods to ensure developmental appropriateness. Although the interview package was developed for young children, the different age groups were involved in this study due to availability of families within the timeframe of this study.

Seventeen children, five parents and one teacher took part. This included five child contributors in individual co‐design sessions (3‐5 year‐olds), three child participants in user‐tester interviews (3‐5 year‐olds) and nine child contributors (6‐12 year‐olds) in the group session. Families were invited through online advertisements on social media (Facebook, *n* = 1 child; Instagram, *n* = 1 child and 1 parent), while no families took part from Twitter (now known as X). The study information was also shared through friends, family and colleagues of the first author, comprising six children, four parents, and one teacher, which ensured a diverse set of contributors and child participants from ethnic minority backgrounds. The first author also contacted a local community centre, where a children's club for 6–12‐year‐olds was selected based on the availability of the children and venue (*n* = 9 children).

The children were ethnically diverse, ranging from White British, British Bangladeshi and British Arab background. One child took part in more than one co‐design phase. Families were predominantly from the North‐East of England, with some from other regions in England taking part online.

Some co‐design sessions took place during the COVID‐19 pandemic and associated restrictions, and thus the sessions were initially conducted online, and later in‐person. Individual in‐person sessions were conducted in the child's home or in the home of a familiar relative, while one group session was conducted at a children's community centre. Each individual co‐design session took place with one child at a time, with one parent present. User‐tester interviews took part either online or in the child participant's home.

Sessions were not audio or video recorded as they were conducted to inform the development of the activities rather than as formal data collection for analysis. Sessions were designed to last between 20 and 40 min. The co‐design activities were undertaken to inform the development of the methods and did not require formal ethical approval. User‐tester interviews served two purposes: to conclude the co‐design process and form the pilot interviews for the wider PhD study. Ethical approval for this specific part of the work was sought and granted by Newcastle University (reference number 02352).

### 3.2 The Co‐Design Process

The materials, activities used, feedback from the co‐design contributors, as well as the researcher's observations and reflexive notes, were all part of the process and data. The co‐design process consisted of four phases divided into the following: Exploration, Adaptation, Confirmation, and Evaluation. This co‐design framework, summarised in Figure 1, was developed for this study. Feedback was mainly sought from the children in each of these phases, yet parents were also invited to provide their opinions regarding their child's engagement with the methods. At the end of their session, each child received an age‐appropriate gift worth £5 (e.g., small toys).

**Figure 1:**
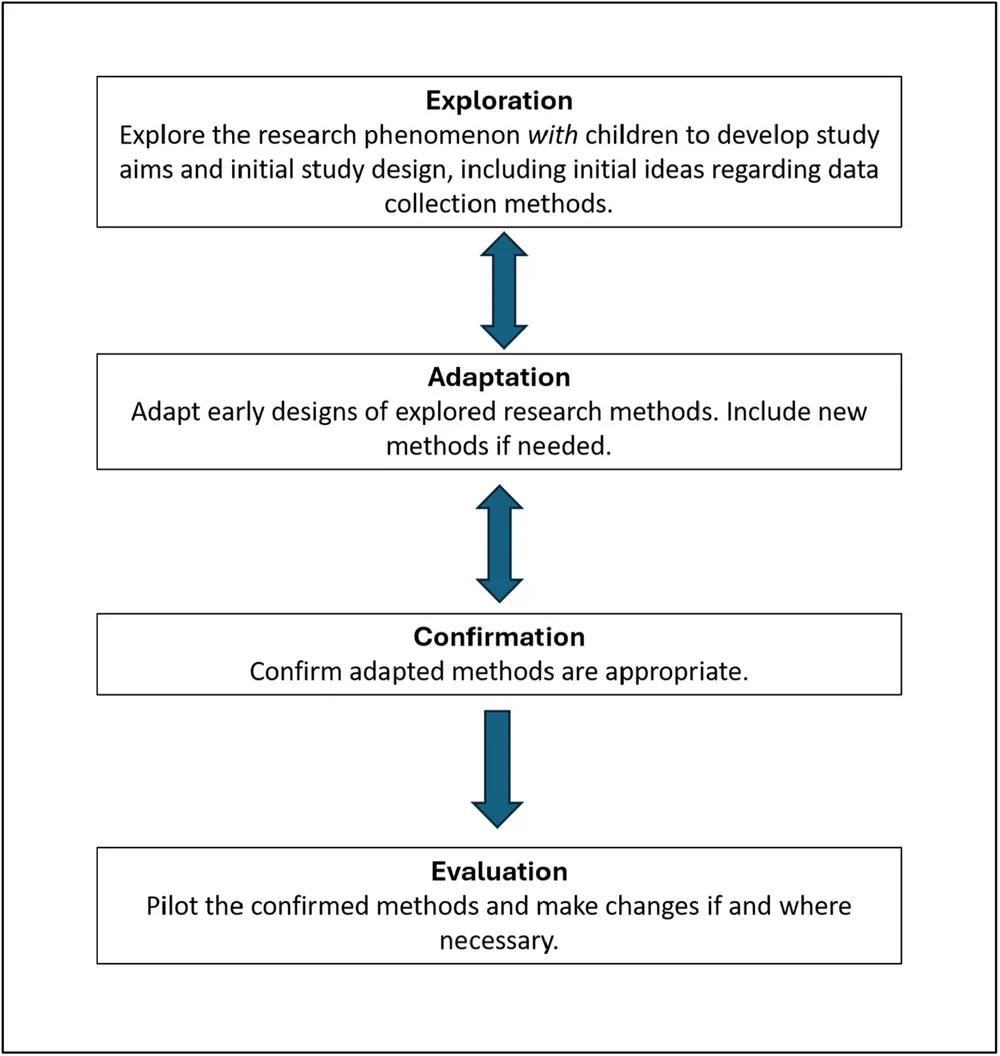
The Co‐Design Framework Developed for this study.

In total, six individual co‐design sessions took place with young child contributors and three user‐testing interviews with child participants. Three children were involved in the Exploration, one of which continued with the Adaptation phase alongside two new children (see Table 1 for summary). These sessions also welcomed observations and comments from parents (*n* = 5), although not all parents provided feedback. One group session with nine children (6‐12 year olds) was conducted during the Confirmation. One teacher also contributed to the Confirmation via email.

**Table 1:** Summary of co‐design sessions.

Co‐design phase	Number of sessions	Number of contributors or participants	Location of activity (n = sessions)	Timeframe
**Exploration**	3 individual sessions	3 child‐contributors (2 parent‐contributor)^a^	Online (1), Familiar relatives home (1) Child's home (1)	October 2021
**Adaptation**	3 individual sessions	3 child‐contributors (2 parent‐contributor)	Online (1), Familiar relatives home (1), Child's home (1)	December 2021 – July 2022
**Confirmation**	1 group session 1 individual session	9 child‐contributors 1 teacher‐contributor	Child community centre (1) Online via email (1)	July 2022
**Evaluation**	3 individual interviews	3 child participants (1 parent‐contributor)	Online (1), Child's home (2)	September 2022

^a^ Each child session involved a parent present, however one parent did not provide feedback in the session, thus they have not been included in this table.

#### 3.2.1 Phase I – Exploration

The research phenomenon was investigated during this phase, wherein the aims and research design were explored in collaboration. Working with children during this phase ensured resources could be invested into a project worthwhile and important within young children's lives [30]. Two image‐based activities were the focus here: pre‐made photographs and child‐led drawing. Pre‐made photographs involved photos from the child's reception year or teacher‐created photo books, facilitating children's *voices* and providing a safe *space* through developmentally appropriate activities. Specifically, this activity was used as memory prompts to support emotional expression and recall of experiences related to the event depicted [31]. Parents were asked to provide these photos, which enabled the discussion to take place either online or in person, as the visual materials were already available and easily shareable.

The second activity involved drawing, conducted in‐person to allow the researcher to observe the drawing and provide resources, although one child chose to use their own resources (e.g., a chalkboard). Reflecting on prior research on school transitions [24, 32], and methods to support children's voices [13, 14], children were asked to draw a self‐portrait of themselves in school, followed by a discussion of their drawing, adopting the ‘Draw and Tell’ method [33].

#### 3.2.2 Phase II – Adaptation

The Adaptation phase focused on working together with young children to further develop the initial methods from the Exploration phase whilst also trialling new methods. In this phase, some sessions combined multiple methods, allowing children to express preferences and identify what they found most enjoyable or engaging. Two versions of picture activities, and two puppet activities were explored.

Expanding on the earlier picture‐related sessions, in this phase, pre‐selected school‐related images were shared with a child as memory prompts. These images consisted of five familiar school settings and activities reflected in classrooms in England (e.g., carpet area, desk space, playground, and school uniforms). Images without facial expressions were selected to avoid influencing the child's interpretations. The session was conducted online during a puppet‐elicited interview with a doll, Rosie. In the online session on Microsoft Teams, images were presented through changing the virtual backgrounds of the researcher, creating the impression that Rosie was exploring different areas of school (see Figure 2). The idea was that Roise had never been to school before, positioning the child as the expert and promoting the child's voice [17]. The researcher asked the child, talking through the doll, to describe the scenes and explain what Rosie might feel in these school environments. Rosie was selected to reflect the participating child's characteristics (e.g., female and South Asian), which may improve the child's engagement in the session [34]. Roise used simple verbal and nonverbal cues (e.g., nodding, smiling), and spoke in short sentences to encourage the child's input while reinforcing her younger age. This dynamic supported age‐appropriate communication [35].

**Figure 2:**
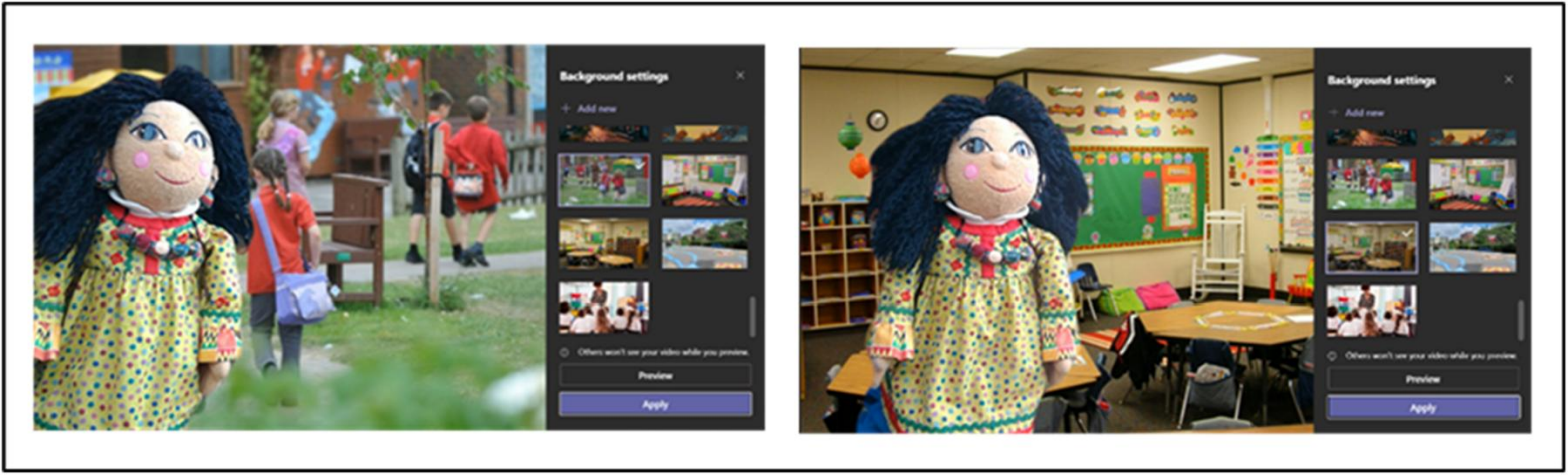
Puppet‐elicited session using pre‐selected images for the background online.

Following this activity, a modified version of the Berkeley Puppet Interview (BPI) [36] was introduced. The BPI consists of two identical puppets sharing statements on a topic, requiring the child to select the puppet they align with [36]. This puppet‐elicited interview was introduced to explore the child's preferred puppet interview technique. Two identical multi‐coloured snake puppets presented opposing statements (e.g., “I like school” vs. “I don't like school”), and the child was asked to select the one that matched their own views.

In a different session with other children, the Pictorial School Stress and Wellbeing Measure (PSSWM) by Murray and Harrison [25] was also trialled. The PSSWM includes four pre‐set questions to gauge the child's perception of challenges in school, such as ‘how does the child in the picture feel?’ [25]. These methods were introduced in the co‐design sessions to gauge its acceptability, for example the child's enjoyment and level of engagement, such as short or long responses from the children.

#### 3.2.3 Phase III – Confirmation

This stage consisted of working together with children to confirm the interview methods and make any final changes based on their suggestions, before evaluating the methods via the Evaluation phase. Research waste, i.e., time and money, can be minimised here by investing in involvement to confirm the appropriateness of a research design to its target population [23]. Ensuring acceptable child‐friendly methods collect the qualitative data, as opposed to unverified methods, could save time as the quality of data collected has not been impacted by inacceptable methods.

A group session with 6‐12‐year‐old children, and an email discussion with a teacher was conducted at this stage. The group session was reserved for the last co‐design phase to collect multiple child opinions at once on the appropriateness of the final methods. After sharing the co‐designed interview methods with them, the children were asked their opinions through an open discussion on their perceptions of taking part in these proposed activities. Following confirmation of the suitability of a puppet‐based activity, the children then worked on designing a puppet that would be suitable for young children, such as appearing friendly and liked by all the children regardless of their characteristics (e.g., gender). The group's recommendations were then used to ensure the puppet used for the interviews represented the characteristics they deemed important for developing a safe interview *space*. For the session with the teacher, communication took place via email. The interview materials and proposed procedures were shared with the teacher, who provided feedback on the suitability of the methods for young children.

#### 3.2.4 Phase IV– Evaluation

The Evaluation phase conducted a practice run on a small scale of all the methods, together, that have gone through the previous phases and deemed acceptable up until this point. This phase could achieve cost‐effectiveness by preventing the use of methods in the study that are not child‐appropriate and tailored to the target populations needs.

Three user‐testing interviews took place with child participants. The children's role was to complete the activities exploring their school experiences. Responses from children concluded the co‐design process through a final confirmation on the ethical considerations ensuring children's *voices* can be captured [16]. Each test round started with a rapport‐building activity with drawings, followed by Murray and Harrison's [25] pictures from their PSSWM as photo‐prompts during a puppet‐elicited interview. The PSSMW [25] screened for ongoing assent, for example, the research would ask before introducing a new image “would you like to see the next picture?”. The images were also selected to achieve the aim of the study, to discuss school‐related challenges in detail.

One of these interviews took place online as the family did not live in the North‐East. At the start of this session, a short game, similar to Simon‐Says, was played with the child and puppet. For the in‐person sessions, rapport‐building began with an A3 sheet showing an incomplete drawing of two school buildings. The activity, framed as completing the school image, was designed to orient the child toward thinking about school. This activity also allowed the researcher to assess verbal comprehension and adjust language accordingly [37] (e.g., adopting child‐led terminology such as ‘big school’).

Informed by earlier feedback from the Confirmation stage, the doll Rosie was replaced with a puppet named Mochi. The concept remained that the puppet had never attended school. Children guided Mochi through each scenario, interpreting the image, describing the characters’ emotions, and suggesting strategies for managing difficult feelings. Throughout the interview, both the puppet and researcher thanked the child for their help, reinforcing rapport through validating the child's contributions [38].

## 4 Results

The outcomes from each co‐design phase are summarised in Table 2 below.

**Table 2:** Outcomes from the co‐design sessions.

	Co‐design phase			
	Exploration	Adaptation	Confirmation	Evaluation
**Drawings**	Dislike self‐portrait drawings, but drawings more generally can help children think about the topic.			Use drawings as rapport activity to orient child about research topic, offering the choice of self‐portraits, and extra paper for child‐led drawing choices.
**Pictures**	Parents may dislike sharing personal photos of their child.	Pre‐made pictures are useful but requires flexible interaction.	Use pre‐made pictures as prompts.	Allow children to decide how to interact with the pre‐made prompt pictures.
**Puppets**		Limited conversation with the child and puppet, restricting data collected.	Puppet should have animal features.	Need to build rapport between child and puppet not just child and researcher.

### 4.1 Evolving Nature of the Drawing Activity

Children commented positively on the activity, particularly enjoying the variety of drawing materials provided, echoing findings from previous studies on the importance of materials in sustaining engagement [14]. Drawings help children express emotions they may not have fully processed or find difficult to articulate [14, 39]. During the early Exploration phase, all parents (*n* = 2) expressed their surprise at their child's ability to describe in detail their school experience, some of which had not previously been known to the parents. The familiar nature of drawing may have contributed to children's willingness to share, offering a comfortable alternative to only verbal expression [14].

A common assumption in child‐centred research is that all children will enjoy supposedly ‘child‐friendly’ methods [12, 14]. In this study, cultural and family‐related considerations emerged that challenged this assumption. A 4‐year‐old South Asian child appeared unfamiliar with the task of drawing a self‐portrait, requesting support from the researcher (e.g., dots to trace) around drawing human features. His parent later explained that drawing people was not a common practice due to their cultural and religious beliefs, consistent with Islamic teachings discouraging depictions of animated figures. Nevertheless, other participating South Asian Muslim children did engage in drawing figures, suggesting that generalisations based on culture and belief systems are insufficient and that flexible, choice‐based activities are essential to uphold children's autonomy and to respect diversity.

Based on these insights, the drawing activity was retained but reframed as a rapport‐building tool, adapted into a more general drawing task rather than a self‐portrait exercise, and no longer treated as a primary method of data collection. Through this adaptation, it avoids compromising children's ability to express themselves (*voice*) and continues to provide an inclusive *space* for children from a Muslim background. In subsequent evaluation sessions, children demonstrated a strong sense of agency during the drawing activity, for example, instructing the researcher on how to contribute to their drawings. Such interactions were interpreted as indicators of the children's empowerment and comfort within the interview context, achieving a safe *space* highlighted within the Lundy model [16]. This also offered an important mechanism to assess non‐verbal cues related to engagement or withdrawal. Given that children entering school are increasingly socialised to follow adult‐led instructions [14, 40], incorporating activities they can control may help disrupt perceived power hierarchies and encourage authentic participation.

### 4.2 The Transition From Child‐Created to Researcher‐Created Pictures

In the early stages of the co‐design process (Exploration phase), contributing children demonstrated the ability to recall detailed school experiences when prompted by images of themselves provided by parents. For instance, one child described a peer conflict that occurred shortly after the image was taken, including how they sought support from a teacher to resolve it. This aligns with prior research suggesting that photographs can evoke emotions and elicit rich details [41]. As the subjects of the images, children appeared motivated to share their stories, and in some cases, nursery photo‐books facilitated the discussion. However, this method was constrained by ethical considerations wherein one parents was uncomfortable sharing personal photos of their child. Practical limitations, such as lack of access to school environments, also constrained the utility of this approach. In several cases, children only narrated events directly linked to the image, restricting the depth and breadth of their expression of their experiences. These limitations highlighted modifications were necessary to avoid overly narrow discussions.

As the co‐design progressed into the Adaptation phase, the use of researcher pre‐made pictures was adopted. These pictures encouraged children to reflect on their broader experiences beyond the events depicted in a single image. In one online session on Microsoft Teams, background images were changed to illustrate different school locations, with the doll, Rosie, positioned in the screen. This immersive technique appeared to capture the child's attention in a manner similar to storybook reading [42]. The presence of Rosie may also have facilitated disclosure through signalling a safe environment [29, 43]. Nonetheless, ethical concerns arose around the possibility that children could misattribute agency to the puppet [29] and forget that the researcher was also still present, potentially leading them to share more than they intended. Additionally, the online format could limit children's ability to exercise control over the session, which reduces the sense of empowerment [18] typically fostered in child‐led activities. The online approach was not carried forward, although the use of pre‐made images was retained.

In subsequent in‐person sessions, the Pictorial School Stress and Wellbeing Measure (PSSWM) [25] was introduced. Following the structured four‐question protocol associated with each image for this measure, children were encouraged to discuss the depicted scenarios. These conversations often revealed emotions previously unknown to their parents, suggesting the method's effectiveness in eliciting meaningful responses. However, the repetitive nature of the format appeared to reduce engagement, with one child asking how many images remained, an issue also observed in prior studies using this protocol [15, 44]. To address this, flexibility was introduced by removing the standardised questions, thereby enabling children to lead the discussion and engage with the images more freely.

During the Evaluation phase, children decided to use leftover drawing materials from the rapport‐building activity to complete the blank faces in the PSSWM images. This unprompted behaviour informed the final design of the method, where drawing was incorporated as an optional response format. This adaptation addressed prior concerns about drawing ability [14] and cultural and religious sensitivity around illustrating faces. In combination with the use of a puppet, these modifications enhanced children's control over their participation and sustained engagement throughout the session. The removal of the rigid questions enabled a more natural conversation, allowing children to describe the scenes in their own words and connect them to personal experiences. Ultimately, these iterative refinements improved the responsiveness of the image‐based interview technique, positioning it as a child‐led, flexible, and ethically sensitive method, achieving the criteria of *voice* within the Lundy [16] model.

### 4.3 Designing the Puppets as Active Social Agents

In all sessions involving puppets, children expressed enjoyment both verbally and behaviourally demonstrated through their level of engagement. Curiosity about the puppet's characteristics and a willingness to share their stories was common. Two parents had initially described their child as ‘shy’ or ‘reserved’ yet were surprised by the degree of interaction that occurred, both with the researcher and the puppet. Aligned with previous findings, puppets can serve as tools for encouraging self‐expression in young children [29, 43], while also challenge assumptions about children's capacities when supported by developmentally appropriate methods.

The specific design and format of puppet‐based interactions influenced the child's responses. For example, in contrast to the session with the doll Rosie, the session involving two identical puppets providing opposing statements (e.g., “I like school” vs. “I don't like school”), elicited less detailed responses. Although the child reported enjoying talking to all puppets in the session, more flexibility was needed to ensure the children's responses were not restricted by the structured interview format.

Further adaptations to puppet design were made following the Confirmation session with older children during a group session. Based on their recommendations, an animal‐like puppet was chosen for its perceived friendliness, gender‐neutral colours and large eyes were identified as features that elicit comfort (see Figure 3). These criteria informed the selection of the final puppet, named Mochi, whose design aligned with the features deemed most appealing and appropriate by the children.

**Figure 3:**
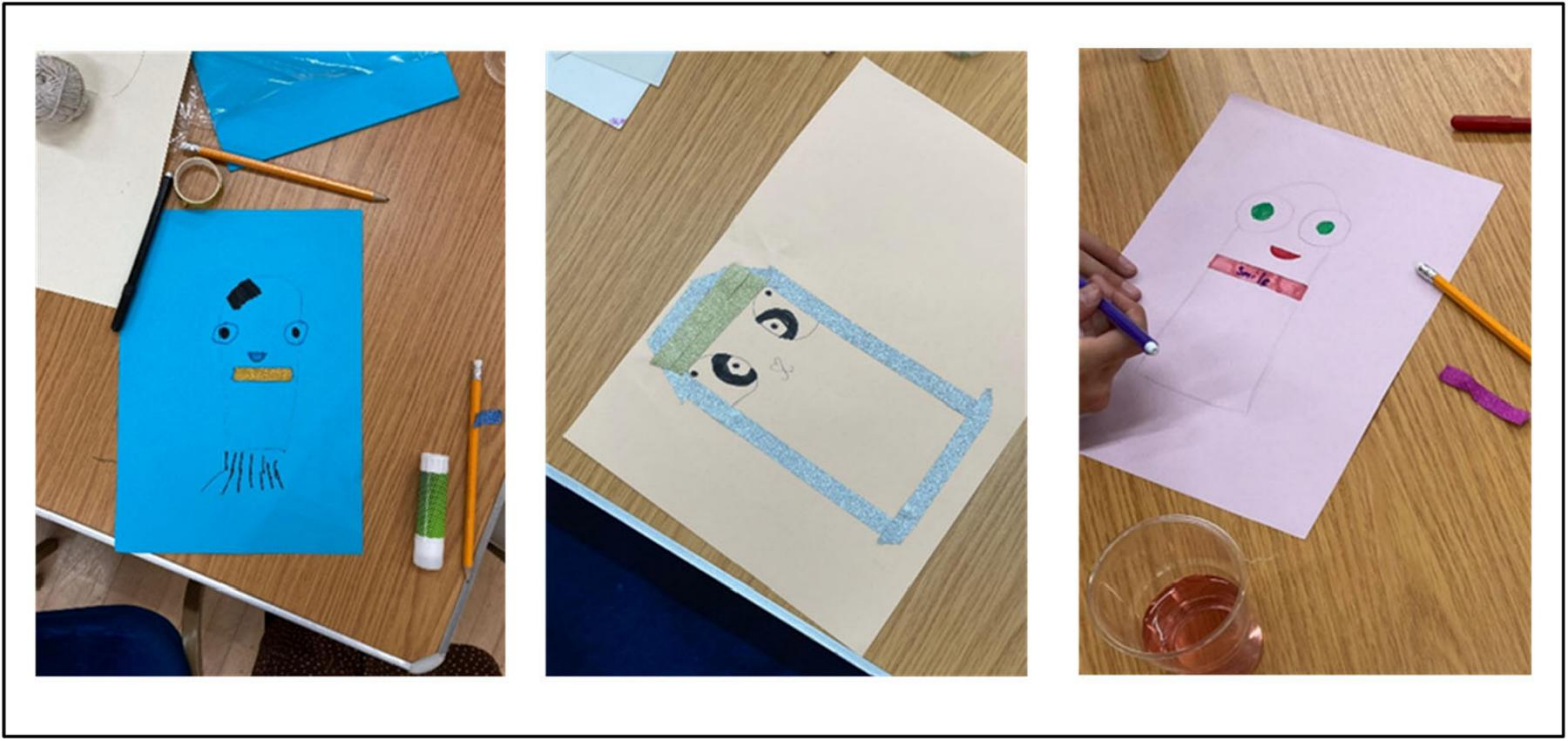
Children's recommendations for the puppet charactristics.

A key insight from the Evaluation phase was that children needed to build rapport not only with the researcher, but with the puppet itself. For instance, one child who had appeared engaged at the start of the session became more reserved when the puppet was introduced later, possibly perceiving the puppet as an independent social agent [43]. This change in behaviour suggested children may view puppets as distinct entities, highlighting the importance of their own connection. It was concluded that the puppet and child engage in their own rapport building activity.

Previous research has documented physical interaction between children and puppets as part of the rapport‐building process (e.g., hugging the puppet) [43, 45]. Drawing from this, children were invited to brush the puppet's hair using their hands, a task that served the functions of reducing the perceived formality of the research setting and satisfying their natural curiosity. The addition of a scripted response from the puppet, such as thanking the child for brushing its hair, was used to reinforce rapport and signal appreciation. This combination of playful interaction and emotional reciprocity positioned the puppet as a friendly ally in the interview [43], achieving both *space* and *voice* [16] through enhancing children's comfort and expression.

### 4.4 Strengths and Limitations of This Study

A key strength of this study was children's recommendations and preferences shaping the methods. Working *with* children emphasised their expertise as co‐designers and ownership of their experiences. Drawings, pictures and puppets are regarded as ‘child‐friendly’ methods, yet this study demonstrated that despite their child‐friendly nature, adaptions to these methods were still required. The children's involvement ensured the qualitative methods facilitated their expression, with ethical considerations in mind, whilst being sensitive to personal characteristics that could hinder their self‐expression via the provided tool.

An additional strength to the study was through including the voices of children from an ethnic minority background. Clavering and McLaughlin [46] argued children's characteristics, such as their ethnicity, influence their life experiences yet ethnic minority children's voices are often missing in participatory research. Rouncefield‐Swales et al [7] noted participatory research may usually involve working with pre‐established groups, wherein children and young people may be more self‐confident and comfortable within research setting than non‐involved children [47]. Recommendations received by these children could omit important considerations from children of an ethnic minority backgrounds or more reserved children. Through their inclusion in this study, a unique consideration regarding the appropriateness of child‐friendly methods was revealed for South Asian Muslim children. Maintaining flexibility in how children interact with these methods is essential to honour their agency, the family's belief systems and culturally‐sensitive practices, upholding child's right to express their views, ideas, and experiences freely in the medium of their choice [1, 16].

A limitation of this co‐design study occurred when gauging children's enjoyment during the Exploration and Adaptation phase by asking direct questions. The sessions were not yet designed to collect feedback through developmentally appropriate approaches, such as inviting children to draw what they had enjoyed and explain why. Adopting child‐appropriate methods to explore their opinions may have provided greater insight into changes needed for these methods. Likewise, drawing activities did not go through all stages of the co‐design process, as other methods were explored and adopted instead. Ensuring this method underwent Adaptation and Confirmation phase with children could have developed a more suitable method. However, the applied drawing activity achieved its aim of orienting the children towards the research topic and fostering a sense of empowerment before the substantial part of the interview. In the future, researchers adopting this co‐design framework may benefit from selecting two or three methods to be trialled out at the first phase to ensure all approaches have undergone each stage, thereby increasing the likelihood of developing child‐friendly methods. Whilst observing the children and collecting their direct feedback can be useful, the researcher could also organise a short activity at the end of the session where children explain why they may have enjoyed or disliked the activity, for example sharing their experience to a puppet who is anticipating the same activity in the future.

The materials were also selected by the researcher rather than developed with children. Involving children in designing the materials (e.g., illustrations on the pre‐made images) could have influenced the final iteration of the methods. Despite this, children were still able to influence the materials to some degree, such as draw faces on the pre‐made images, affording them the ability to express themselves in a way that suited them. Moreover, feedback from the 6‐12 year old cohort may have differed from the 3‐5 year old children. However, at least two young children provided feedback on the methods at each co‐design phase before the Confirmation phase, ensuring that their perspectives informed the development of the interview package.

Finally, there are multiple versions of participatory methods, such as puppets controlled by children [48], that may have engaged children more effectively but were not explored in this study. Future researchers could develop these methods and materials with young children which may offer different levels of engagement.

## 5 Conclusion

Young children's involvement as co‐designers or research participants is often limited [8], yet this study demonstrates young children's capabilities as valuable contributors to research. The interview methods have been designed to be implemented with limited resources and without connections to additional gatekeepers (e.g., schools), providing an advantage for researchers with limited time and resources. Whilst this interview package was specifically designed for a study exploring the experiences of 4–5‐year‐olds experiencing the transition to formal education, the broader context was to develop something that other researchers could to tailor (e.g., change the pictures) for their own research topic. Key learning from this study can be applied to any iterations of these methods, such as building rapport between child and puppet. Collaborating with children has ensured developmentally appropriate methods have been achieved, implementing Lundy's [16] recommendation for safe *spaces* and children's *voices*, thus managing ethical considerations.

## Author Contributions

**Arwa Katab:** conceptualisation, investigation, writing – original draft, writing – review and editing, visualisation, methodology, formal analysis, data curation, resources, project administration. **Bronia Arnott:** conceptualisation, funding acquisition, methodology, writing – review and editing, supervision. **Timothy Cheetham:** conceptualisation, supervision, writing – review and editing, formal analysis. **Olivia Craw:** conceptualisation, writing – review and editing, supervision. **Niina Kolehmainen:** formal analysis, supervision, conceptualisation, methodology, writing – review and editing, writing – original draft.

## Ethics Statement

The initial discussions and meetings with children did not require ethics approval, the latter testing of materials had Newcastle University ethics approval (reference number 02352).

## Patient Consent Statement

Parents provided verbal and/or written consent for both their child and their own participation – verbal consent for the initial discussions, and verbal and written consent for the subsequent user testing of materials; children provided verbal assent for all participation. No identifiable data were collected for the co‐design study.

## Conflicts of Interest

The authors declare no conflicts of interest.

## Data Availability

The data that supports the findings are available in the findings of this manuscript.
